# What Is the Impact of Nutrition Literacy Interventions on Children’s Food Habits and Nutrition Security? A Scoping Review of the Literature

**DOI:** 10.3390/ijerph19073839

**Published:** 2022-03-24

**Authors:** Beatrice Velpini, Gabriele Vaccaro, Virginia Vettori, Chiara Lorini, Guglielmo Bonaccorsi

**Affiliations:** 1 School of Specialization in Hygiene and Preventive Medicine, University of Florence, 50134 Florence, Italy; beatrice.velpini@unifi.it (B.V.); gabriele.vaccaro@unifi.it (G.V.); 2 Department of Health Science, University of Florence, 50134 Florence, Italy; chiara.lorini@unifi.it (C.L.); guglielmo.bonaccorsi@unifi.it (G.B.)

**Keywords:** nutrition literacy, nutrition security, food habits, intervention

## Abstract

Introduction: Nutrition literacy is a relatively new concept that seems to have a relevant role on the quality of people’s diets; however, we do not know the role of nutrition literacy interventions on children’s food habits and nutrition security. Methods: We conducted a literature review on four databases (PubMed, Embase, Web of Science, and Cochrane) and we considered studies describing the impact of interventions aimed at improving the quality of children’s diet. Results: A total of six articles were finally included. The total number of participants included in the studies was 4016, and the median study size was equal to 224 participants. Studies included in the review reflected a broad heterogeneity of interventions. Conclusions: The results were contrasting and revealed that the most effective type of nutrition literacy interventions included the following characteristics: technological components, involvement with multiple modalities, duration of more than 4 weeks, and face to face sessions.

## 1. Introduction

The concept of nutrition literacy (NL) can be described as a set of individual and context-related characteristics which allow adherence to a healthy diet that respects the guidelines on proper nutrition [1]. 

In our previous conceptualization [1], NL was considered as part of the multifaceted and broader concept of food and nutrition literacy (FNL), which embraces several aspects regarding individual knowledge and competency, interactive skills, and specific characteristics of the food environment. A food- and nutrition-literate community is realized when people are able to eat food ensuring their nutritional health from the perspective of a sustainable food system [1]. FNL includes several useful knowledge areas, skills, and abilities, and also reflects people’s motivation to adopt appropriate behaviors and to make healthy food choices for themselves as well as for others. Additionally, the concept of FNL also refers to the characteristics of the environment in which people live that can favor or neglect the adoption of healthy choices and behaviors. In fact, environment can influence the realization of initiatives to promote a healthy lifestyle, the ability of professionals to interact with their community and patients, and the ease of understanding food labels, all contributing to appropriate and aware choices.

From this point of view, an individual’s FNL evolves over time and is a context-dependent dimension, exerting an important role in all stages of life. From the literature, the concept of FNL seems to be interconnected with that of food and nutrition security (FNS) [1].

In the developed countries, the condition of FNS is determined by both the availability of safe, nutritious, economic, and tasty food, as well as by individuals’ possibility and capacity to access and use it [2,3]. In this regard, some authors have discussed the relationship between FNL and FNS, because it seems that consumers’ food choices, influenced by their knowledge and competence in the field of food and nutrition, could play a determinant role in guaranteeing adequate diet for the whole population [4].

A connection between the two concepts has been well documented in the recent literature. Cullen et al. [5] highlighted that food behaviors, skills, and the condition of security in the field of food, cannot be separated because they are part of the same construct—that is, FNL. According to this perspective, Gallegos [6] included FNS in the dimension of FNL with other specific skills, knowledge, and socio-cultural aspects: all these elements impact on an individual’s capacity to choose, prepare, and store food items to maintain the quality of their diet across time [7]. This is in accordance with another research, which emphasizes the importance of FNL to address the challenge of the so-called “food paradox” [8]. This issue can be summarized as “while people are becoming more interested in food, they are actually becoming more disconnected from it” [9], and confirms the separation between people and the food they consume. Nowadays, television programs regarding food and, more generally, information about foodstuffs have increased beyond proportion. On the contrary, consumers’ time spent cooking, growing, or eating food is steadily falling [9]. Additionally, the overproduction and overselling of foodstuffs as well as low adherence to plant-based diets are adverse characteristics of the current food supply chains and behaviors, and have a negative impact on the environment as well as animal welfare and human health, general society, economy, and culture [10]. Considering these perspectives, Block et al. [8] attributed a central role to FNL: it can deeply influence human health and well-being, through educational food programs and initiatives that inform people and spread individual knowledge, motivation, and ability, in addition to providing the opportunity to apply that knowledge with the aim to use food adequately.

Within the concept of FNL, the part mostly related to nutrition is NL, which focuses mainly on the selection of foods to be included in the diet on the basis of their nutritional properties, and on the availability of food products on the market.

In a national context, the population level of NL, both in adults and young people, has been very little explored; an exception is represented by the study of Tabacchi et al. [11] that evaluated FNL during the process of validation of a five-domain toolkit ‘preschool-FLAT’ targeted at 3–6-year-old children. However, there are no data that explore the association between NL and the condition of FNS.

Considering these premises, we decided to conduct a scoping review aimed at investigate the potential impact of NL interventions on FNS. Considering NL interventions, we referred to food and nutrition education programs or training, or the distribution of informative documents, and we focused on how these promotive actions have affected access to and capacity to use food in order to adhere to a healthy diet.

## 2. Materials and Methods

**Figure 1:**
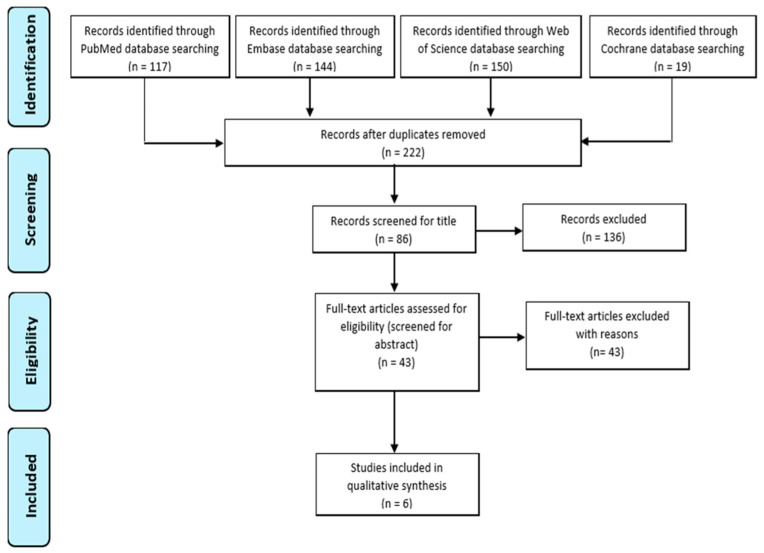
Preferred Reporting Items for Systematic Reviews (PRISMA) flow diagram for the scoping review process.

Systematic research of the literature was conducted following the methodology and guidelines for the development of a systematic scoping review [12]. A flowchart, produced according to the Preferred Reporting Items for Systematic Reviews (PRISMA), documents the selection process of the articles to be included in the final review (Figure 1).

We used the combination of the words “nutrition” and “literacy” in the search string and explored four databases (PubMed, Embase, Web of Science, and Cochrane). We limited our research to articles published in English, adopting no temporal limits. 

We included articles where the following criteria were retrieved: dealing with NL interventions consisting of nutrition education programs or training, or the distribution of informative documents (including e-learning and e-docs). We focused on children (including new-borns) and/or adolescents (0–17 aged). We only considered studies that described the impact of these interventions on improving the quality of young people’s diet. This scoping review was performed during May and June 2021 and it was independently performed by two authors (B.V. and G.V.). The same two authors screened the full text of the six papers included in the final synthesis and collected, from the selected studies included in the synthesis, the following elements: a description of the interventions, nutrition health outcomes influenced by the interventions, and how the outcomes had been measured. In case of disagreement, a third author (V.V.) intervened to discuss and clarify the discrepancies with the two reviewers (B.V. and G.V.).

The search provided N = 466 records. We did not manually search for other items, considering this number as sufficient to answer the research question.

After removing duplicates, the remaining number of records was N = 212. We reviewed the title and abstract of all these records, and excluded N = 179 papers because they were not related to the topic of the research; then, we focused on N = 43 items that were reviewed as full text. We excluded N = 32 papers because they did not explain or discuss the impact of the NL interventions on FNS and food habits; finally, we excluded N = 5 articles because they involved adults. The final number of included papers was six, which specifically examined the relationship between the two concepts in children or adolescents. 

We extracted data from papers by considering several fields, as suggested by the guidance developed by the Joanna Briggs Institute members [12]: title; author(s); year of publication; source origin/country of origin; aims/purpose; study population and sample size (if applicable); methodology; intervention type and comparator (if available); the outcomes measured; and the method for assessing those outcomes. In addition, we checked the articles included in the final synthesis, revised the text of the papers, and traced the description of how NL interventions influenced the condition of nutrition security or food habits. By tracing the outcomes of the interventions in this way, we obtained a greater understanding of how NL influences the condition of FNS.

## 3. Results

A total of six articles are included in this scoping review [13,14,15,16,17,18]. The total number of participants included in the studies was 4016 and the median study size was equal to 224 participants. An overview of these studies, including participant characteristics, study size, intervention details, assessment measures, and study findings, is provided in Table 1. 

Countries where studies were conducted included: USA (*n* = 2), UK (*n* = 1), Iran (*n* = 1), Turkey (*n* = 1), and Indonesia (*n* = 1). A total of three studies came from countries (Iran, Turkey, and Indonesia) that are currently classified as developing countries by the Development Assistance Committee (DAC).

The recent focus on NL is confirmed by the fact that all the studies considered were published since 2018. The studies included in the review reflected a broad heterogeneity of interventions: face to face training, class education and class simulation, in-person sessions with technology components, teaching laboratory through science engagement, and a classroom-based curriculum. Two [17,18] of the six interventions were devoted to infants (0–36 months) through mothers, and the rest to adolescents (11–17 years). The duration of interventions ranged from 4 to 16 weeks and outcomes were measured at two time points: at baseline and after the intervention. The timing of the evaluation of the effect of the intervention ranged from immediately post intervention to 1 year later.

Four studies [15,16,17,18] used a controlled trial design to compare outcomes of the intervention group to those of the control group. Two [14,16] of the six studies were pilot versions of food and nutrition education programs, and in one of these [14] incentives were provided to encourage participation, in the form of a fitness tracker, weekly take-home bags packed with a food item, and a $5 gift card for each session attended.

The studies included in our synthesis were based on different conceptualizations of literacy in the field of nutrition: three [13,16,17] referred to the degree to which people can process and understand basic nutrition information; one [14] dealt with the ability to use food knowledge and skills to make healthy dietary choices and encompassed aspects of planning and managing, selecting, preparing and eating healthy foods; and another [15] referred to the knowledge, skills, understanding and confidence to use health and care information and services and to apply these to lifestyle choices.

The food habits/food consumption were assessed with different measurement tools, including the Adolescent Food Habits Checklist (AFHC), a food consumption survey, a food frequency questionnaire on the consumption of 15 specific food items, self-report items from the Centers for Disease Control and Prevention (CDC)’s Youth Risk Behavior Surveillance System (YRBSS), and a 10-item food frequency and amount questionnaire (FAQ). There was also a broad heterogeneity of nutrition knowledge measurements that included the Adolescent Nutrition Literacy Scale (ANLS), a knowledge, attitude, and behavior (KAB) survey, a 17-item survey and an 18-item multiple-choice visual format questionnaire.

Two [13,14] of the six included studies reported a negative change from baseline to post intervention. Kalkan et al. [13] showed a decrease in the scores at the ANLS and at the AFHC scores between the baseline and the measure after the intervention. Particularly, these results seem to be attributable to the increasing difficulty after the training of the ability to change, share, and discuss food and nutrition information [19] and the ability to critically judge information on food and nutrition [19]. This could be explained by the fact that the intervention was too short (2 h each week for 4 weeks). Wickham et al. [14] showed a decrease in the consumption of fruits, vegetables, and water and an increase in the consumption of sugar-added beverages. Instead, Harley et al. [16], obtained positive change through a classroom-based curriculum intervention, highlighting a greater improvement in fruit and vegetable and vegetable-only consumption over the 6-week intervention period compared to the control group, and significant changes in nutrition knowledge.

Sirajuddin et al. [17] showed that maternal NL significantly influenced the status of stunting measured as Height for Age (HAZ) in the intervention group represented by 43 mothers. Specifically, class education, class simulation, home visits two times a month, and a total of 15 visits for child growth monitoring were performed. Researchers note that in the intervention group there was a significantly greater reduction (9.3%) in stunted children than in the control group (2.4%) after a 3-month intervention. Seyyedi et al. [18] observed positive intervention effects on wasting indicator status among undernourished children under 3 years of age using the WHO growth indicators HAZ, WHZ (Weight-for-Height Z-Score) and WAZ (Weight-for-Age Z-Score). Indeed, the mothers in the intervention group showed greater improvement with regard to the ability to critically analyze information about food and nutrition, nutritional knowledge, attitudes towards feeding, and nutrition practice. Additionally, the children in the smartphone group showed greater progress with regard to wasting status, underweight and stunting status compared to the control group.

## 4. Discussion

NL dimension could have a relevant role in influencing FNS, taking into account that individual knowledge and competence are important for proper diet management. Our goal was to explore the literature to identify which NL interventions affect FNS condition and their characteristics. We focused on a younger segment of the population whose diet habits are crucial for their future nutritional status. From this literature review, it seems to emerge that, in general, studies revealing children and adolescents’ NL levels are few, although in a few cases the impact of NL interventions on the quality of diet and FNS conditions was explored (consumption of fruits, vegetables, whole grains and sugar-added beverages; children’s stunting status, wasting and underweight). Our review brings out contrasting results about the impact of NL interventions on the FNS of children and adolescents.

Harley et al. [16] seem to indicate an improvement in adolescents’ eating habits involved in the consumption of fruit, vegetables, and whole grains through sessions including lessons and recipes; this result is in line with what was proposed by other authors [20,21], who found a direct proportionality in the scores detected with the Adolescent NL Scale (ANLS) and the Adolescent Food Habit Checklist (AFHC) tool.

An increase in the consumption of vegetables and fruit was also found in the study of Wickham et al. [14] which, despite the intrinsic limits of a pilot study, showed an improvement in the adolescents recruited through various educational methods. In this article, knowledge about nutrition also increased, while the consumption of sugar-added beverages was not positively influenced by literacy in the field of food and nutrition. In fact, as suggested by El-Ahmady et al. [22], knowledge alone cannot improve habits without the contribution of other factors: knowledge alone will not drive a person to practice a habit unless he or she believes in its concept. Thereby, nutrition consciousness must be developed [23]. NL, however, beyond the food choices adopted, is useful to improve awareness of one’s diet, as shown by Woods-Townsend et al. [15], where adolescents subjected to teaching laboratories tended to judge their lifestyle more severely than the control group. 

The relationship between NL and food habits was highlighted in Kalkan et al. [13], where the scores related to the dimension of NL and quality of diet decreased, with a significant positive correlation, after a face-to-face training intervention. Probably, as the authors themselves point out, the decrease is due to excessive shortness of the education intervention, hypothesizing that an intervention of longer duration would have had a different outcome. Moreover, face-to-face lessons did not seem to be not very effective, while more involving methods, for example workshops or multimedia activities, appeared more profitable, as demonstrated by the study of Harley et al. [16], in which face-to-face lessons were combined with an experience of cooking lessons, and results proved to be better. The relationship between literacy and habits was also shown by Khorramrouz et al.’s [24] research, in which, through the use of HFI (Household Food Insecurity) and FNLIT (Food and Nutrition Literacy) scales, children who had a more severe condition of FNS in terms of insufficient food quality and insufficient food intake, also obtained worse scores to the measurement of NL. 

The tools used in interventions on adolescents were limited to theoretical lectures and the administration of questionnaires and multimedia activities; among these, multimedia activities obtained better results which were more in line with the designated objectives. Dealing with adolescents, it would probably be advisable to suggest, for further studies, methodologies of didactic games that are not limited to intellectual components, but also involve emotional and socializing elements; moreover, education through images should be exploited, especially in order to guarantee both immediate and long-lasting attitudes.

Nutritional literacy interventions on mothers could be a good starting point for improving children’s nutritional status condition. From results collected through our systematic review, it seems maternal NL interventions significantly influenced children’s stunting status [17,18], wasting, and underweight [18]. According to the literature, children living in families with a limited access to nutritionally adequate and safe food or with an inability to acquire foods in socially acceptable ways were more likely to have a lower overall nutritional knowledge, food choice literacy, and food label literacy [24]. Despite the small number of cases examined, the positive effect of nutritional literacy interventions on FNS was more evident in children than in adolescents. This can be explained by the fact that interventions on adolescents, which have their primary outcome in the change in food habits, are contrasted by social pressure and social norms that influence their eating behaviors [25,26]. Consequently, nutritional literacy is only one determinant of adolescents’ health, and their nutritional habits reflect culture and family traditions more than literacy, and they are often hard to change. Another reason why interventions in adolescents are less effective may be that the change in eating habits was assessed through the completion of questionnaires that could have led adolescents to answer in a distracted and casual way [13,14], affecting the reliability of the results when compared to the objective assessment of children’s growth status.

As shown by our review, number of participants, type of intervention, ways to evaluate, and subjects of interventions (nutritional terminology, healthy eating, diet-related chronic diseases, and healthy food selection) were very heterogeneous. All these aspects limit the comparability of the studies included in the final synthesis, and the possibility to converge towards conclusive results.

Our results about the impact of nutrition literacy interventions on nutrition security status and food habits are contrasting, and this is due to several factors. As admitted by authors of the included articles, the results of the interventions were not markedly positive; therefore, the possible reasons leading to these results should be deeply investigated in every part (purposes, methods, time, selection of population, measurement of results). It must be considered, however, that interventions on adolescents rarely achieve immediate effects, especially when trying to modify habits embedded in familiar and social contexts. Finally, considering that interventions have been structured with rather varied methodologies and implemented in very different geographical and cultural areas, it is also difficult to identify, at this moment, which elements of the studies constituted strengths to be preserved and reinforced, and which were weaknesses to be modified. However, these studies provide basic elements to start appropriate research pathways.

The scope of this review was limited to literacy interventions affecting eating habits and nutritional security; therefore, purely educational interventions were not included. Few nutritional literacy interventions identified in this review focused on FNS, and most were based on dietary behaviors, making it more difficult to draw conclusions about the effectiveness of nutritional literacy interventions. In addition, as already specified, the investigated samples were different in terms of age (children and adolescents), sample size, and dimension of geographic area, reducing the generalizability of the results.

## 5. Conclusions

NL interventions to improve nutritional security and food habits show to be promising in terms of a positive impact on children and adolescents’ diet behaviors. The most effective type of intervention seems to be that including technological components, involving multiple modalities, with a duration greater than 4 weeks, and using face-to-face sessions. In order to reinforce this evidence, future studies need to be carried out with higher methodological quality. In addition, we found a gap in the literature about the role of social determinants on young people’s quality of diet. This limited our comprehension of the relationship between nutrition literacy and food habits and nutrition security for this specific age group, taking into account that young people could be nutritionally insecure owing to everyday life circumstances, such as bad socio-economic conditions.

Although the results are contrasting, the studies included in this review represent a first attempt to analyze how FNS could be improved through NL. Our systematic review, regardless of its impact on knowledge, has highlighted the research opportunities existing in this area. Therefore, this is a first step in research which, hopefully, can be useful for those who intend to go on with studies in this field. 

We would encourage future discussion to focus on the role played by young people’s living context on their level of NL and, consequently, on their quality of diet.

## Figures and Tables

**Table 1:** Participant characteristics, study size, education intervention details, assessment measures, and study findings.

Reference, Year of Publication, Country of Origin	Aim/ Purpose	Study Population/Sample Size	Study Design	Intervention	Outcomes Measured	How Outcomes Are Measured	Results
Kalkan et al. [13] 2020 Turkey	To study NL level and food habits; to investigate the effect of a short nutrition training program on both.	N = 200 high school students.	Cross-sectional study.	Nutritional face to face training by the research team members (8 h–4 weeks).	NL and food habits.	ANLS and AFHC were applied before and 3 months after the training.	Negative results: the scores at the ANLS and at the AFHC decreased. Positive correlation between ANLS pre-test and post-test scores with AFHC pre-test and post-test scores.
Wickham et al. [14] 2018 USA	To form an advisory group (Kid Council) to direct the design of a food literacy program; to implement a pilot version of the program to assess participants’ attitudes to participate.	N = 9 adolescents (11–15 years old) with access to a computer and a cellphone and able to read and speak English.	Cross-sectional study.	Six in-person sessions (1 h) and technology components (fitness tracker, text messages, a companion website) with focus on ability to select fruit and vegetables, choose water over sugar-sweetened beverages, and engaged in physical activity.	Knowledge, attitude, and behavior for fruit and vegetable intake, sugar-sweetened beverages, and physical activity.	A KAB survey and a food consumption survey at the first and last session.	Knowledge remained low at postsurvey (with an increased trend from baseline). Attitudes toward vegetables increased slightly. Attitudes toward sugar-added beverages increased. Behavior questions related to fruit, water/sugar-added beverages, and physical activity increased; vegetable behavior scores decreased. The consumption of fruit, vegetables, and water decreased and the consumption of sugar-added beverages increased.
Woods-Townsend et al. [15] 2021 England	To evaluate whether taking part in LifeLab improved adolescents’ nutrition and health literacy, and whether participation changed how they viewed their own health behavior.	N = 2929 adolescents (13–14 years old) from secondary schools/academies.	Cluster-randomized controlled trial.	Teaching laboratory dedicated to improving adolescent health through science engagement.	Theoretical health literacy score and lifestyle perceptions.	Questionnaires were administered to control and intervention participants at baseline and again 12 months later.	Adolescents in the intervention group showed a greater improvement in the theoretical literacy score and they tended to judge their lifestyles to be less healthy than the adolescents in the control group.
Harley et al. [16] 2018 USA	To examine the effectiveness of YCA to impact healthy eating.	N = 248 middle school-aged students (11–13 years).	Nonequivalent control group design.	YCA, a classroom-based, hands-on culinary and NL curriculum with 62-h sessions.	Changes in times per day of (F)/(V), and WG consumption; vegetable and WG preferences; self-efficacy for cooking, tasting new foods, and eating, servings of F/V per day; student engagement; readiness to increase F/V consumption and nutrition knowledge.	Survey administered 1 week prior to the first session and 7 weeks after the sixth session.	Significant increases in times per day of F/V consumption in the intervention group compared to the control group. Increases in WG consumption showed a trend toward significance. Student engagement and nutrition knowledge showed significant intervention effects.
Sirajuddin et al. [17] 2021 Indonesia	To analyze the effect of maternal nutritional literacy intervention on stunting in infants.	N = 85 mothers with children aged 0 to 6 months.	Randomized control trial.	Class education, class simulation, home visits twice a month and total visits of 15 times for child growth monitoring and hand sanitation.	Distribution of nutritional status and distribution of stunting.	HAZ	There was no difference in the distribution of nutritional status, but MNL significantly influenced the status of stunting in the intervention group.
Seyyedi et al. [18] 2020 Iran	To evaluate the support of nutritional education delivered by a smartphone application on undernourished preschoolers.	N = 110 mother–child (0–36 months) pairs	Randomized control trial.	Smartphone application and educational content with an interactive healthy-child care guide structured into a set of learning topics.	Changing in wasting status and in the mothers’ nutritional literacy (critical knowledge, feeding attitudes, and nutritional practice).	WHZ indicator for wasting status, WAZ indicator for underweight status, HAZ indicator for stunting status and an instrument to measure nutritional literacy (based on WHO recommendations) before and after the 6-month intervention period.	The mothers in the smartphone group showed greater improvement with regard to the nutritional literacy dimensions: critical nutritional knowledge, attitudes towards feeding, and nutrition practice. The children in the smartphone group showed greater progress with regard to the wasting status, underweight and stunting status.

Legend of the abbreviations that appear in the table: Nutrition Literacy (NL); Adolescent Nutrition Literacy Scale (ANLS); Adolescent Food Habits Checklist (AFHC); Knowledge, Attitude, Behavior (KAB); Youth Chef Academy (YCA); fruit/vegetable (F)/(V); whole grain (WG); Height for Age (HAZ); Maternal Nutrition Literacy (MNL); Weight-for-Height Z-Score (WHZ; Weight-for-Age Z-Score (WAZ); World Health Organization (WHO).

## Data Availability

Not applicable.
